# Effect of pH on the Formation, Disintegration and Antioxidant Activity of Mung Bean Protein Fibrils

**DOI:** 10.3390/antiox14121399

**Published:** 2025-11-25

**Authors:** Yike Tian, Shuning Zhang, Weining Huang, Ning Li, Li Liang

**Affiliations:** 1 State Key Lab of Food Science and Resources, School of Food Science and Technology, Jiangnan University, Wuxi 214122, China; 2 Guangzhou Puratos Food Co., Ltd., Guangzhou 511400, China

**Keywords:** mung bean protein, fibrillation, aggregation, antioxidant activity

## Abstract

Protein fibrils are highly ordered structures. Mung bean protein (MBP) fibrils were fabricated after heating under acidic condition. The formation mechanism, structural variety and antioxidant capacities of mung bean protein fibrils were investigated under different pH conditions. The fibrillation process was characterized by extensive hydrolysis of MBP into low-molecular-weight peptides, which subsequently self-assembled into fibrils with increasing contour length over time. The formed fibrils exhibited a dominant β-sheet structure but demonstrated high sensitivity to pH changes. Upon adjustment to pH 7.0, the fibrils changed into amorphous aggregates, accompanied by a structural transition to random coils. Pretreatment of MBP with neutral protease and chymotrypsin yielded hydrolysates with distinct peptide profiles, leading to the formation of fibrils with varied morphologies upon acidic heating. Furthermore, the antioxidant activity of MBP and its fibrillar aggregates was highly dependent on pH and structural state. While fibril formation at pH 2.0 led to a reduction in radical scavenging activity of MBP, the subsequent pH-shifted aggregates at pH 7.0 resulted in a significant enhancement of antioxidant capacity. These findings provide fundamental insights into the formation, stability, and bioactive properties of MBP fibrils, emphasizing their pH-dependent behavior.

## 1. Introduction

Protein fibrils are highly ordered structures with nanoscale diameters, micrometer lengths and high aspect ratios. Food proteins can assemble to form amyloid fibrils, which change their functional properties and bioactivities [1]. Protein fibrils mechanically prevent the thin film between adjacent air bubbles or oil droplets from rupturing, thereby enhancing foam and emulsion stabilities in comparison with native proteins [2,3]. However, the negative impact of fibrillation on functional properties has also been reported for the foaming properties of egg white protein and the emulsion properties of oat globulins and β-lactoglobulin [4,5]. Due to the conformational modification of proteins and/or peptides released by hydrolysis in the process of fibrillation, some protein fibrils could be regarded as potential antioxidants [6]. The increase in the antioxidant activity of oat globulin and whey protein was reported during their fibrillation [5,7]. The acidic thermal treatment enhanced the Fe^3+^ reducing capacity of β-lactoglobulin [8]. The antioxidant activity of fibrillated and hydrolyzed rice protein was found to be 2.09 times greater than that of native rice protein to stabilize oil-in-water emulsions [9]. Therefore, research on the fibrillation of proteins is necessary to provide a more profound understanding of their processability and nutritional properties.

The fibrillation of legume proteins exhibited diverse morphological and kinetic behaviors, which were primarily governed by protein composition and hydrolysis as well as the external conditions [2]. The linear fibrils of β-lactoglobulin could be observed at both acidic and neutral conditions, but were absent at the protein isoelectric point [10]. However, soybean protein fibrils were only observed at acidic pH and changed into amorphous particles or aggregates upon adjustment to neutral and alkaline pH values [11]. The fibrils from chickpea protein were typically straighter than those from cowpea protein [12]. A critical step in fibril formation was protein unfolding and hydrolysis. Soy protein fibrils formed by protein unfolding, hydrolysis and assembly from an irregular spherical structure to a coiled, intertwined thread-like structure during hydrolytic heating. However, the disintegration of soy protein fibrils occurred upon overheating [3]. During heating at acidic pH, panda bean protein isolate was progressively hydrolyzed into shorter fractions that participate in forming fibrils with both flexible and rigid morphologies [13]. When pea protein was pretreated using ultrasound and trypsin, its fibrils with trypsin pretreatment were longer than the control and those with ultrasound pretreatment [14]. The types and characteristics of the released polypeptides might determine the self-assembly pathway.

Mung bean protein possesses abundant essential amino acids, exceeding the FAO/WHO recommendation for adults [15]. Mung bean protein is also a potential source of bioactive peptides. The peptides obtained from mung bean protein displayed considerable antioxidant capacity, anti-hemolytic activity and angiotensin-I converting enzyme inhibitory activity [16,17,18]. To broaden the application scope of mung bean protein in food and its related industries, fibrillation as a modification is being investigated for functional optimization. Mung bean protein exhibited a higher potential in fibril formation compared to the proteins from red kidney bean and red bean [19]. For cowpea protein and mung bean protein, the slender prefibrillar assemblies with worm-like morphology underwent progressive twisting and assembly to form multi-stranded amyloid fibrils with thickened diameters as the ionic strength increased [20]. The fibril formation capacity increased by purifying mung bean protein to 8S, and the fibrillated mung bean protein at pH 4–8 demonstrated better foaming properties than the proteins without fibrillation [21,22]. However, there is no report about the impact of mung bean protein fibrillation on its antioxidant activity.

In this study, the formation and disintegration mechanism of mung bean protein fibrils and their characterization along with antioxidant activity were investigated at pH 2.0 and 7.0. Herein, the acidic condition was employed for fibril formation, while the neutral condition was used to evaluate the structural stability and behavior of the fibrils. The formation and disintegration of MBP fibrils were observed through an optical microscope, transmission electron microscope (TEM), atomic force microscope (AFM), circular dichroism (CD) and sodium dodecyl sulfate-polyacrylamide gel electrophoresis (SDS-PAGE), while the characterization of MBP and fibrils at various pH was determined by ζ-potential and surface hydrophobicity. Finally, the antioxidant activity was evaluated by examining the scavenging activities of ABTS and DPPH radicals.

## 2. Materials and Methods

### 2.1. Materials

Mung bean protein was obtained from Zhongtai Biotechnology Co., Ltd. (Jingzhong, Shanxi, China). 8-Anilino-1-naphthalenesulfonic acid (ANS), 2,2-diphenyl-1-prcrylhyrazyl (DPPH) and 2,2′-azino-bis(3-ethylbenz-thiazoline-6-sulfonic acid) (ABTS) were bought from Sinopharm Chemical Reagent Co., Ltd. (Shanghai, China). Mica used was originated from India. Neutral protease (~100,000 U/mL) and chymotrypsin (1500 U/mg) were bought from Jiangsu Boyang Bioproducts Co., Ltd. (Nantong, Jiangsu, China) and Hefei Bomei Biotechnology Co, Ltd. (Hefei, Anhui, China), respectively.

### 2.2. Sample Preparation

Mung bean protein at 2–7% was dissolved in Milli-Q water, and their pH values were around 8.8. The solution of 2% mung bean protein was noted as MBP. After the aqueous solution of 3% mung bean protein was adjusted to pH 7.0, stirred for 2 h, and then centrifuged at 10,000× *g* for 30 min, the supernatant was noted as MBP-7. Moreover, the aqueous solution of 3% mung bean protein was adjusted to pH 2.0 with 6 M HCl, stirred for 2 h, then stored at 4 °C overnight. After the acidified protein solution was centrifuged at 10,000× *g* for 30 min, the resulting supernatant was filtered through a 0.45-μm membrane. The final protein concentration in the supernatant was 2%, which was measured by the Biuret method, and the sample was denoted as MBP-2. MBP-27 was obtained by adjusting MBP-2 to pH 7.0.

The dispersion of 7% mung bean protein was adjusted to pH 7.0, heated to 50 °C, mixed with neutral protease at an enzyme activity of 3000 U/g of protein for 4 h. In addition, the dispersion of 4% mung bean protein was heated to 37 °C, adjusted to 8.0 and mixed with chymotrypsin at an enzyme activity of 3000 U/g for 0.5 h. These hydrolysis reactions were terminated by enzyme inactivation at 90 °C for 10 min. After cooling the hydrolysates to room temperature, their pH was adjusted to 7.0 and centrifuged at 10,000× *g* for 30 min, and the supernatants were collected and lyophilized. The hydrolyzed products obtained by neutral protease and chymotrypsin were re-dissolved in Milli-Q water to obtain a final concentration of 2%, marked as MBHn and MBH_C_.

The solution of 2% MBP-2 was heated at 85 °C for 12 h and named MF-2. The solutions of 2% MBHn and MBH_C_ at pH 2.0 were also heated at 85 °C for 12 h [19,21]. Aliquots were collected hourly and immediately transferred to an ice bath to quench the reaction. All fibrils were stored at 4 °C. MA-7 was obtained by adjusting MF-2 to pH 7.0.

### 2.3. Optical Microscopy

A drop of MF-2 solution was placed on a microscope slide for observation at Polarizing microscope (M320P-HK830, Shenzhen AOSVI Optical Instrument Co., Ltd., Shenzhen, China) with a 4× objective IN bright-field mode. The scale bars were added by Image J 1.50i.

### 2.4. Transmission Electron Microscopy (TEM)

TEM (HT-7800, Hitachi, Tokyo, Japan) was utilized for morphological observation at the accelerating voltage of 80 kV. MF-2 was diluted to 0.5 mg/mL with Milli-Q water adjusted to pH 2.0. A 10 μL aliquot of the solution was dropped on a carbon-coated copper grid and dried at room temperature.

### 2.5. Atomic Force Microscopy (AFM) Measurements

Samples were diluted to a protein concentration of 0.01 mg/mL by Milli-Q water at pH 2.0. Exactly 10 μL of the diluted solutions were deposited on freshly cleaved mica surface and dried in a clean and sealed container overnight. AFM images were obtained in the tapping mode, with the height information being acquired through NanoScope Analysis v1.9 software. The counter length of fibrils formed after 4, 8, 12 h were analyzed, and 40 random fibrils were taken for each sample [23]. AFM data processing and statistical analysis were conducted with FiberApp software v2.1.

### 2.6. Surface Hydrophobicity

ANS at 8 mM was dissolved in 10 mM phosphate buffer and stored at 4 °C in the dark. Sample solutions were diluted to a protein concentration of 1 mg/mL in Milli-Q water at corresponding pH values. After 0.4–2 mL of diluted samples were mixed with 20 μL of ANS solution and incubated in the dark for 20 min, the mixture was diluted to 4 mL with Milli-Q water. Fluorescence intensity of ANS was measured using a spectrofluorometer at an excitation wavelength of 390 nm and an emission wavelength of 470 nm. Surface hydrophobicity (H_0_) was quantified as the initial slope value of the fluorescence intensity versus protein concentration plot [24].

### 2.7. ζ-Potential

Samples were diluted into Milli-Q water at correspondingly pH values with 6 M NaOH or 6 M HCl. The ζ-potential values were ascertained by NanoBrooker Omni Particle Sizer and ζ-Potential Analyzer (Brookhaven Instruments Ltd., New York, NY, USA) with a scattering angle of 90° at 25 °C. ζ-Potential was obtained by applying the Smoluchowski model.

### 2.8. Sodium Dodecyl Sulfate-Polyacrylamide Gel Electrophoresis (SDS-PAGE)

SDS-PAGE was performed with 12% separating gel and 5% stacking gel on Mini-Protean system (Bio-Rad Corp., Richmond, CA, USA). Samples were diluted to a protein concentration of 2 mg/mL with Milli-Q water. Exactly 500 μL of samples were mixed with 500 μL of loading buffer consisting 20% glycerol, 4% SDS, 0.025% Bromophenol blue, 0.12 M Tris-buffer at pH 6.8 and heated in boiling water for 5 min. Exactly 10% of 2-mercaptoethanol was added in loading buffer for reducing condition. The gel was stained by 0.1% Coomassie brilliant blue (R-250) and visualized with ChemiDoc MP imaging system m (Bio-Rad Corp., Richmond, CA, USA).

### 2.9. Circular Dichroism

Samples were diluted to a protein concentration of 0.15 mg/mL with Milli-Q water at corresponding values. The circular dichroisic (CD) spectra of the diluted samples in a 0.1 mm quartz tube were recorded from 190 to 260 nm on using Chirascan V100 (Applied Photophysics Ltd., Leatherhead, UK) [21].

### 2.10. ABTS and DPPH Radical Scavenging Activity

Antioxidant activity was analyzed using ABTS and DPPH assays [5]. The ABTS stock solution was prepared by mixing equal volumes of 7 mM ABTS and 4.9 mM potassium persulfate followed by incubating in the dark for 12–16 h. The ABTS working solution was prepared by diluting its stock solution with Milli-Q water at corresponding pH values to achieve an absorbance of 0.7 at 734 nm. Exactly 100 μL of the diluted samples at 0.5% reacted with 4.0 mL of the working solution for 10 min, and the absorbance was measured at 734 nm. For DPPH assay, the diluted samples at 5 mg/mL were adjusted to pH 2.0 or 7.0 and then mixed with 0.1 mM DPPH in ethanol in a volume ratio of 1:1. After these mixtures stood in darkness for 30 min, the absorbance was measured at 517 nm. Due to sediment formation after pH adjustment, the mixtures of MA-7 or MBP-27 mixed with radical working solution were centrifuged at 6000× *g* for 10 min, and their supernatants were used to determine the absorbance. The mixture of 100 μL Milli-Q water with the radical working solution was set as negative controls, while the samples in 4.0 mL Milli-Q water without the radical working solution were set as blank controls. The radical scavenging activity was calculated using the following equation:

(1) $$Scavengingactivity\left(\%\right)=\left(1-\frac{A_{sample}-A_{blank}}{A_{negative}}\right)\times 100\%$$

where *A*_sample_ was the absorbance of samples with radical solution, *A*_blank_ was the absorbance of the blank controls, and *A*_negative_ was the absorbance of the negative control.

### 2.11. Statistical Analysis

Data of each treatment condition in all measurements were presented as means ± standard deviation for three independent replicates (*n* = 3). IBM SPSS statistics 27 software package (IBM, Armonk, NY, USA) was used for significance analysis (*p* < 0.05) of differences.

## 3. Results and Discussion

### 3.1. Formation of Mung Bean Protein Fibrils

**Figure 1:**
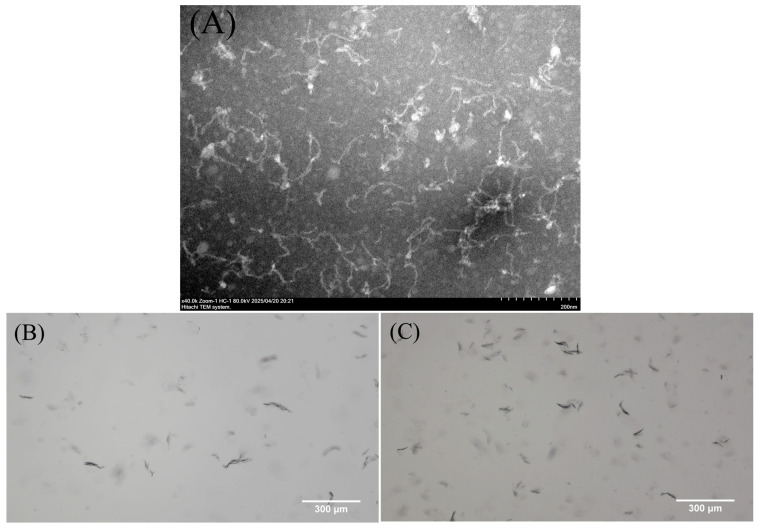
TEM (**A**) and optical microscopy (**B**) of mung bean protein fibrils (MF-2) obtained after heating at pH 2 for 12 h. (**C**) The optical microscopy of MF-2 after storage at room temperature for 7 days.

In the initial experimental attempts, it was found that gelation occurred when mung bean protein concentrations were between 3% and 6% (Figure S1). Therefore, the concentration of mung bean protein at 2% was used for the fibrillation study. After mung bean protein was heated at pH 2 for 12 h, its long fibrils could be clearly observed in TEM and optical microscopy (Figure 1A,B). The fibrils (MF-2) were stable after storage at room temperature for 7 days (Figure 1C).

**Figure 2:**
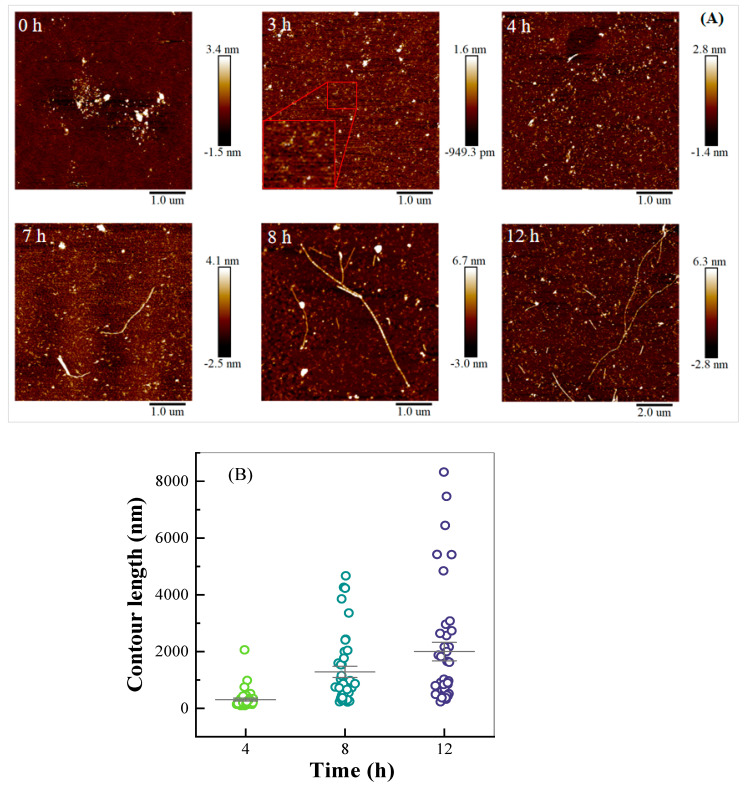
AFM images (**A**) and contour length distributions. The red frame in the 3 h panel outlines the area where nanoscale fibril-like structure is first observed. (**B**) of mung bean protein fibrils after various heating times.

As shown in Figure 2A, the aggregates of mung bean protein were observed with a wide range of sizes at pH 2, possibly due to the dissociation of large aggregates upon acidification. Nanoscale fibril-like structures appeared after heating for 3 h. The formation of short and straight amyloid-like fibrils was detected after 4 h. Branching of long amyloid fibrils was visible after heating for 8 h. Fibril length increased with extended heating time. Similar trends have been reported for fibril formation from legume proteins [12,25,26]. The contour length of mung bean protein fibrils formed after heating for 4, 8, and 12 h was shown in Figure 2B. The average contour length of fibrils grew from 310 nm to 2000 nm as the heating time increased from 4 to 12 h. The longest fibril could reach up to 8320 nm after 12 h. Specifically, the mature fibril system after 12 h of heating comprised both long and short fibrils, with the latter significantly predominating in quantity.

### 3.2. Characterization of Mung Bean Protein and Its Fibrils at Various pH

**Figure 3:**
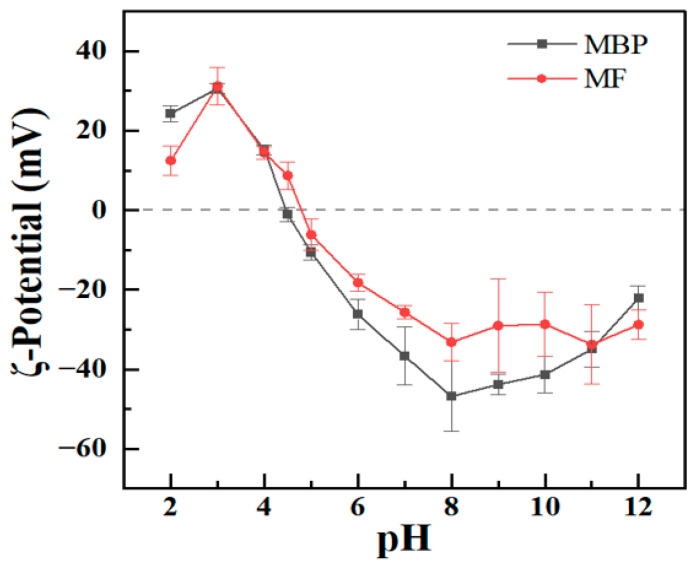
ζ-Potential of mung bean protein (MBP) and its fibrils (MF-2) as a function of pH. Values are presented as mean ± standard deviation (n = 3).

ζ-Potential is widely recognized as an indicator of particle surface charges and serves as a critical parameter for measuring the stability of protein systems [27]. At pH 2, MF-2 and MBP had ζ-potential values of +13 and +24 mV (Figure 3), respectively. These ζ-potential values increased to about +31 mV at pH 3. The ζ-potential values were lower at pH 2 than at pH 3, which may attributed to the hydrolysis of glutamine and/or asparagine into glutamic acid and/or aspartic acid at pH 2.0, as previously reported for mung bean protein and hemp seed protein [28,29]. It was also reported that the ζ-potential values of fibrils were lower than those the non-amyloid materials, because the exposed residues induced by protein hydrolysis and unfolding were concealed during fibrillation [30,31]. Upon further increasing the pH from 3, the ζ-potential values of both MF-2 and MBP began to decrease with pH (Figure 3). The isoelectric points (pI) of MBP and MF-2 were near pH 4.5 and 4.7, respectively. The ζ-potential values of both MF-2 and MBP changed to negative values at higher pH. The ζ-potential values of MF-2 gradually decreased to −26 mV at pH 7. The ζ-potential value of MF-2 reached about −30 mV at pH 8 and then remained constant at higher pH values (Figure 3). However, the ζ-potential values of MBP gradually decreased to −48 mV at pH 8 and then increased to −22 mV at pH 12. The absolute values of MF-2 were lower than those of MBP at pH 2 and across the pH range of 5–10, which was consistent with the results of soybean protein fibrils [31].

**Figure 4:**
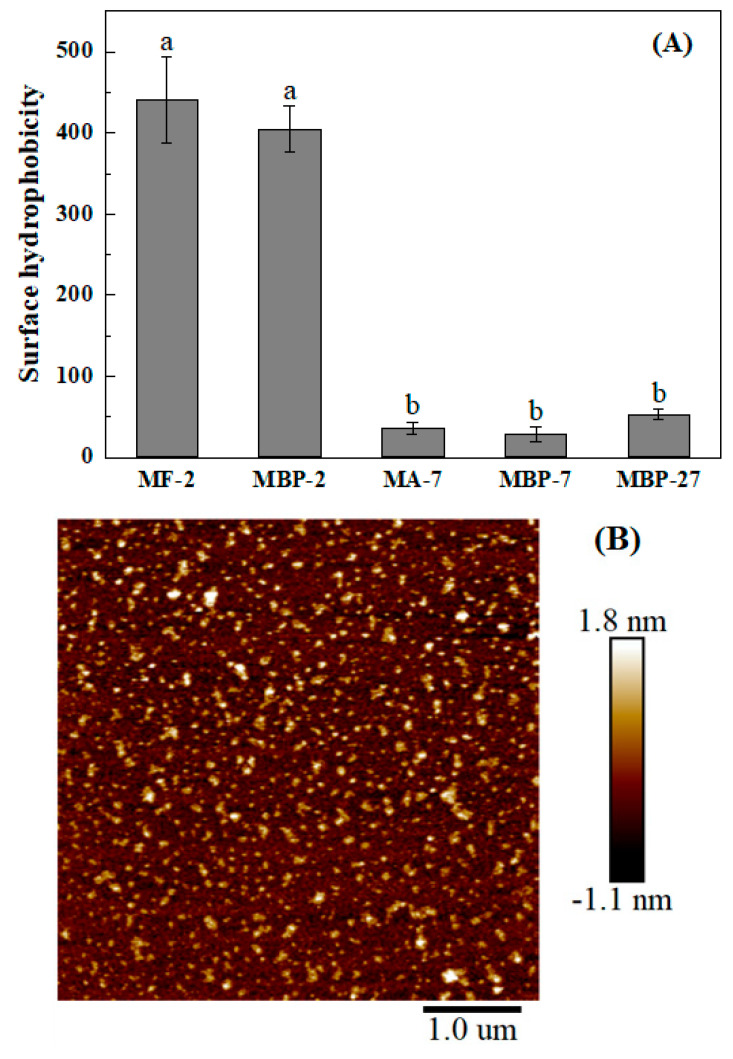
(**A**) Surface hydrophobicity of mung bean protein and its fibrils (MF-2) at pH 2.0 and 7.0. MBP-27 is pH-shifted MBP by acidification to pH 2.0 followed by neutralization to pH 7.0. Different lowercase letters (a,b) indicate significant differences (p < 0.05) among different samples. (**B**) AFM image of MBP aggregates obtained by adjusting MF-2.0 to pH 7.0 (MA-7). Values are presented as mean ± standard deviation (n = 3).

ANS is extensively employed as a fluorescent probe for surface hydrophobicity. Its fluorescence intensity increased as the polarity of surrounding environments decreased [32]. At pH 2, MBP-2 and its fibrils had similar surface hydrophobicity (Figure 4A). When the MF-2 solution was adjusted to pH 7.0, the fibrils disappeared, and large aggregates were observed (MA-7, Figure 4B). Bromelain hydrolysis at pH 7.0 generated thermal-induced fibrillar aggregates from quinoa protein isolate [33]. At pH 7.0, MA-7 also had similar hydrophobicity to MBP-7 and pH-shifted MBP-27. All samples at pH 7.0 had lower surface hydrophobicity than those at pH 2.0, which is negatively correlated with their absolute ζ-potential values (Figure 3). Moreover, the more pronounced aggregation (Figure 2 and Figure 4B) resulted in the burial of more hydrophobic residues, thus lowering surface hydrophobicity.

### 3.3. Formation and Disintegration Mechanism of Mung Bean Protein Fibrils

#### 3.3.1. Secondary Structure and Hydrolysis

**Figure 5:**
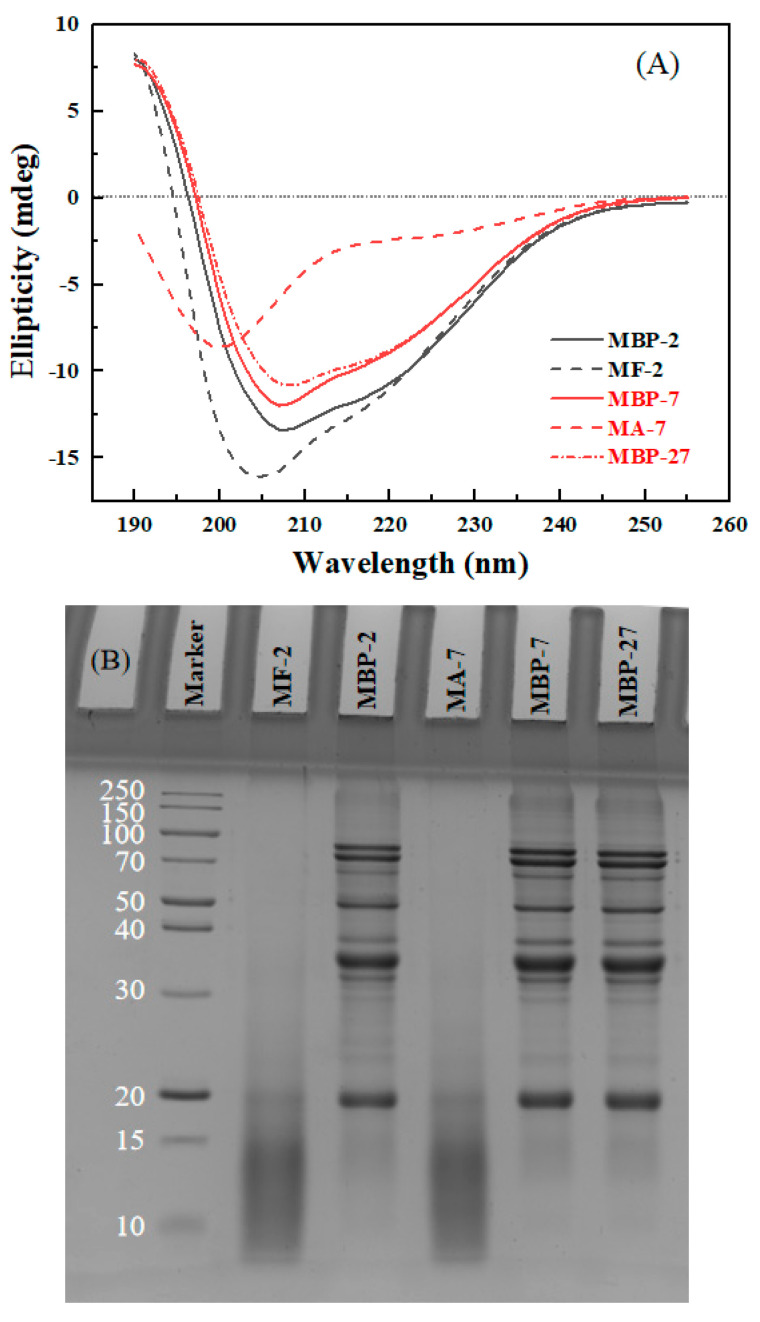
CD spectra (**A**) and SDS-PAGE (**B**) of mung bean protein (MBP) at pH 7 (MBP-7), pH 2 (MBP-2) and pH-shifted from 2 to 7 (MBP-27) and of MBP fibrils obtained by heating at pH 2 and 85 °C for 12 h (MF-2) and MBP fibrils at pH 7 (MA-7).

The secondary structure of MBP and its fibrils under various pH conditions was detected using circular dichroism. The spectrum of MBP-7 showed two negative peaks around 207 and 222 nm (Figure 5A), suggesting that α-helix was the main secondary structure. Both MBP-7 and MBP-2 showed similar spectra, but the ellipticities of MBP-2 were greater than those of MBP-7. The difference may be due to the dissociation of MBP at pH 2.0 (Figure 2), which disrupted the hydrogen bonds and electrostatic interactions, resulting in the alterations of secondary structures [34]. The ellipticity at 207 nm in the spectrum of MBP-27 was a bit less than that in the spectrum of MBP-7 (Figure 5A), suggesting that the difference caused by pH-shifting was limited at the same final pH condition of 7.0. The minimum ellipticities of MF-2 shifted to 204 and 217 nm, illustrating the decrease in α-helix content and the increase of β-sheet and random coil. β-Sheet was recognized as the dominant structure of amyloid fibrils [35]. The spectrum of MA-7 exhibited the minimal peak at 200 nm (Figure 5A), indicating that its predominant secondary structure was random coil. The rise in pH from 2.0 to 7.0 caused destruction of the β-sheet, resulting in the disintegration of fibril structure. The results are consistent with purified soy protein fibrils at pH 11 [11].

MBP-7 and MBP-2 showed the bands around 84, 73, 62, 46, 38, 34, 32 and 18 kDa (Figure 5B). Under reducing conditions, 8S vicilin has four subunits of 26, 32, 48, 60 kDa, 7S globulin has two subunits of 16, 28 kDa, and the subunits of 11S legumin are of 24 and 40 kDa [36]. The pH-shifting treatment from 2.0 to 7.0 did not affect the protein bands (Figure 5B). However, all native protein bands disappeared, and small peptide fractions with molecular weight lower than 20 kDa were only detected in MF-2 and MA-7, confirming the extensive hydrolysis of MBP components during acidic heating treatment. The hydrolysis of soybean protein was reportedly the predominant behavior for its fibrillation, and the formation of polypeptides facilitated to the assembly of various polymorphic forms of amyloid-like fibrils from soybean [3,37]. It is speculated that small peptides become the building blocks of MBP fibrils (Figure 2). Therefore, the fibrillation process of mung bean protein includes the following chronological phases: the destruction of aggregates and hydrolysis of peptide chains, the initiation of nucleation via basic fractions to form initial aggregates as the fibril core, the recognition and attachment of existing cores and units to form short and worm-like fibrils, and the subsequent prolonging and maturation of the fibrils [38].

#### 3.3.2. Fibrillation of Enzyme-Hydrolyzed Mung Bean Protein

**Figure 6:**
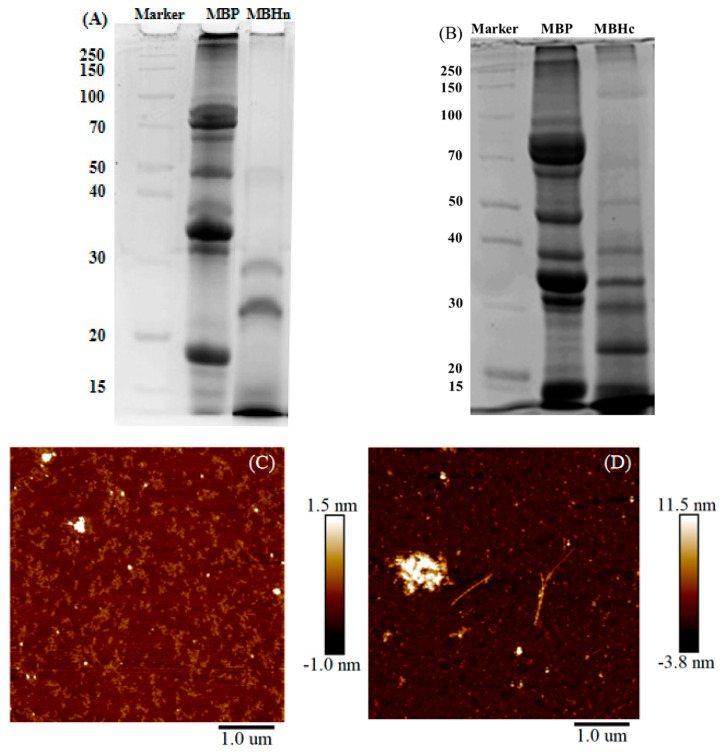
SDS-PAGE of MBHn (**A**) and MBHc (**B**) and their respective AFM images after heating at pH 2.0 (**C**,**D**).

It was reported that the proteolysis pretreatment showed influence on the fibrillation process by impacting the morphology and the structure of fibrils [39]. The rice protein fibrils formed after the pretreatment with alkaline protease possessed a longer contour length than native protein but with a lower conversion rate [40]. In this study, MBP was subjected to hydrolysis by neutral protease and chymotrypsin to investigate the fibrillation of its enzymatic hydrolysates. Chymotrypsin, a serine protease, preferentially cleaves peptide bonds carboxyl-terminal to aromatic and hydrophobic amino acid residues (e.g., Phe, Tyr, and Trp). However, it can also hydrolyze bonds after diverse non-aromatic residues, albeit with lower preference [41]. In contrast, neutral protease, as a metalloprotease exhibiting mild catalytic activity, had no specific enzymatic site [42]. The enzyme type influenced the molecular weight and amino acid composition of hydrolysates. It was found that rice dreg protein pretreated by neutrase released more hydrophobic groups than Alcalase, Flavorzyme, Protamex and trypsin [43]. Soybean protein hydrolysates generated by Alcalase and neutral protease revealed similar N-terminal residue profiles, predominantly comprising Phe, Leu, and Val, but the hydrolysates from neutral protease had an elevated proportion of His [44]. The hydrolyzed products of MBP by neutral protease (MBH_n_) showed emerging bands at the vicinity of 30, 24, and below 15 kDa, along with the decline of the original bands of MBP (Figure 6A). MBH_n_ showed short and curly fibrils after acidic thermal treatment (Figure 6C). For the hydrolyzed products of MBP by chymotrypsin (MBH_c_), the bands emerged at 22 and 15 kDa, bands larger than 40 kDa were barely detected, and the intensity of bands at 40, 35, and 30 kDa was significantly decreased (Figure 6B), compared to those of MBP. MBH_c_ formed long and straight fibrils along with some immense aggregates after acidic thermal treatment (Figure 6D). It was found that β-LG fibrils formed by discrepant building blocks, such as intact denatured protein at pH 3.5 and peptides at pH 2.0, due to different extents of hydrolysis [30]. MBH_n_ and MBH_c_ formed fibrils with different morphologies (Figure 6C,D), due to their different degrees of hydrolysis (Figure 6A,B). After selective proteolysis of β-conglycinin in soy proteins, the fibrils were observed to be semiflexible and long rather than the worm-like fibrils observed in the control [45]. The hydrolysis by Corolase N reduced the fibrillation potency of whey protein isolate and formed fibrils with less homogeneous length distribution [46]. The fibrils formed by rice protein pretreated by alkaline protease showed a higher contour length with a reduced conversion rate [40]. It is thus speculated that the proteins that are either non-hydrolyzed or excessively hydrolyzed are not conducive to forming long fibrils. The length of MBH_c_ long fibrils (Figure 6D) was less than that of MF-2 long fibrils (Figure 2), possibly due to that the combination of chymotrypsin and acidic thermal treatment might cause more pronounced hydrolysis of MBP. Therefore, it is suggested that the diverse morphologies of MF-2 fibrils (Figure 2) resulted from the broad molecular weight of hydrolyzed products obtained through direct acidic thermal treatment (Figure 5B).

### 3.4. Antioxidant Activity

**Figure 7:**
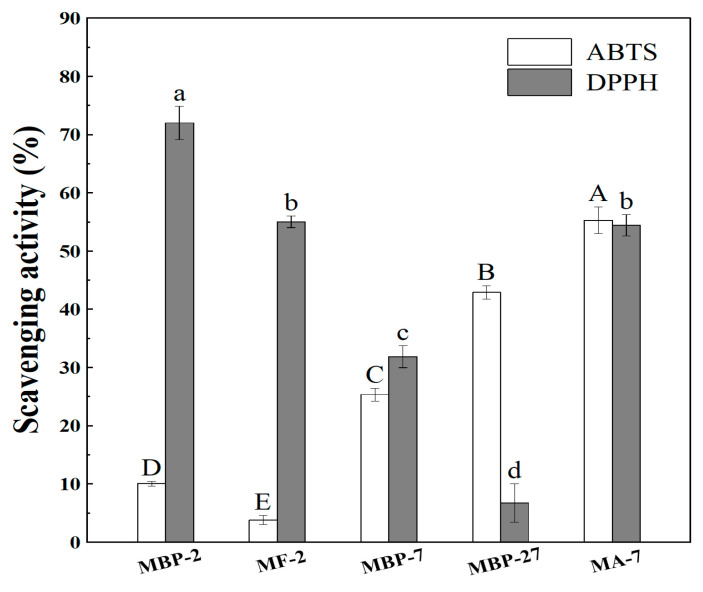
Antioxidant activity of mung bean protein at pH 7 (MBP-7), pH 2 (MBP-2) and pH-shifted from 2 to 7 (MBP-27), of protein fibrils obtained by heating at pH 2 and 85 °C for 12 h (MF-2) and of protein aggregates at pH 7 (MA-7). Different lowercase letters (a–d) indicate significant differences (*p* < 0.05) among different samples within the DPPH assay. Different capital letters (A–E) indicate significant differences (*p* < 0.05) among different samples within the ABTS assay.

Antioxidant activity was analyzed using ABTS and DPPH assays. The ABTS assay was measured in an aqueous environment. The ABTS radical scavenging activities of MBP-2 and MF-2 were 10.05% and 3.79% (Figure 7), respectively. The lower scavenging capacity of MF-2 than MBP-2 (Figure 7) supports that the exposed residues induced by protein hydrolysis and unfolding (Figure 5) were concealed during fibrillation (Figure 2). The ABTS radical scavenging activities were greater at pH 7.0 than at pH 2.0 (Figure 7). The two most popular methods for quenching radicals are HAT (hydrogen atom transfer) and SET (single electron transfer), which possess tremendous differences when exposed to various conditions [47]. HAT is significantly hindered by diffusion, which is greatly facilitated by water and impeded by hydrogen-bonding solvents such as alcohols. In contrast, the rate of electron transfer is not limited by diffusion, and it increases with pH and the extent of ionization [48]. ABTS radicals carry a positive charge [49]. The pH shift from 2.0 to 7.0 causes the deprotonation of carboxyl groups, thereby enhancing the electron donation to the cationic ABTS radical, which resulted in an increase in scavenging activities. The ABTS radical scavenging activities of MBP-7, MBP-27, and MA-7 were 25.34%, 42.90%, and 55.27%, respectively. These results suggest that the exposure of originally encapsulated residues to the aqueous phase was more pronounced by fibrillation combined with pH shifting than pH shifting alone.

The DPPH assay was analyzed in the water-ethanol mixture. MBP-2 and MF-2 scavenged 72.00% and 55.03% of DPPH radicals, respectively. The DPPH radicals are nitrogen-centered with significant steric hinderance due to its bulky molecular structure. The DPPH· reaction rate is determined by the steric accessibility of the radical sites [50]. The steric accessibility of DPPH radicals might be a major determinant of the reaction for MF-2. Instead of the chemical properties of antioxidant compounds, the steric accessibility of the radical site determined the DPPH reaction rate [50]. Conversely, the DPPH radical scavenging activity of MBP was greater at pH 2.0 than at 7.0. In the DPPH radical scavenging assay, antioxidants engage with DPPH radicals through three pathways without exclusion: SET, HAT, and the sequential proton loss electron transfer mechanism (SPLET) [49]. The proton donor changed from -NH_2_ in alkaline solutions to –NH_3_^+^ in acidic solutions, which might lower the energy barrier in deprotonation, facilitating hydrogen donation to the neutral DPPH radicals [50,51]. Precipitation was observed for MBP-27 upon mixing with the DPPH radical ethanolic solution, due to the salt introduction through pH-shifting treatment. This is the reason for the lower scavenging capacity of MBP-27 compared to MBP-7 (Figure 7). The precipitation of MBP-27 reduced its interaction with the DPPH radical, thereby complicating the direct comparison of its scavenging capacity with other samples. However, MA-7 displayed a higher antioxidant capacity than MBP-27 and MBP-7. The DPPH radical scavenging capacities of MF-2 and MA-7 were similar. It is possible that the positive effect of protein conformational modification and hydrolysis counteracts the negative effect of pH shifting. However, another responsible explanation could be the various radical scavenging mechanisms between proteins before and after fibrillation, which showed different responses to pH adjustment. In 10 mM phosphate buffer at pH 7, the DPPH and ABTS radical scavenging capacities of oat globulin and whey protein isolates increased upon fibrillation [5,7]. However, for whey protein isolate fibrils, the fibrillation process displayed no change in the DPPH assay, but significantly increased the ABTS assay when measured at pH 2 [1]. It could thus be concluded that the influence of fibrillation on antioxidant activities showed significant discrepancies at different pH levels. The different solvent system of DPPH and ABTS assays influences protein solubility, radical accessibility, and the predominant reaction mechanism, contributing to the divergent pH responses observed between the two assays [50,52].

## 4. Conclusions

Mung bean protein fibrils were successfully fabricated after heating under an acidic condition of pH 2.0. The fibrillation process was characterized by extensive hydrolysis of MBP into low-molecular-weight peptides, which subsequently self-assembled into fibrils with increasing contour length over time. The formed fibrils exhibited a dominant β-sheet structure. Upon adjustment to pH 7.0, the fibrils changed into amorphous aggregates, accompanied by a structural transition to random coils. Pretreatment of MBP with neutral protease and chymotrypsin yielded hydrolysates with distinct peptide profiles which, in turn, led to the formation of fibrils with varied morphologies. This underscored the critical role of hydrolysis extent and peptide composition in determining fibrillation and the final fibril structure. Furthermore, the antioxidant activity of MBP and its fibrillar aggregates was highly dependent on pH and structural state. While fibril formation at pH 2.0 led to a reduction in radical scavenging activity of MBP, the subsequent pH-shifted aggregates at pH 7.0 resulted in a significant enhancement of antioxidant capacity. These findings provide fundamental insights into the formation, stability, and functional properties of MBP fibrils, emphasizing their pH-dependent behavior. Further studies should consider the optimization of pH conditions to modulate the structure and functionality of MBP fibrils. Additionally, complementary assessments of their nutritional values are highly valuable in evaluating the potential of MBP fibrils in food-related applications.

## Data Availability

The original contributions presented in this study are included in the article/Supplementary Materials. Further inquiries can be directed to the corresponding authors.

## Supplementary Materials

The following supporting information can be downloaded at: https://www.mdpi.com/article/10.3390/antiox14121399/s1, Figure S1: Visual observation of 2% (top) and 6% (down) MBP at pH 2.0 during heating treatment.
