# Supplementary figures and images for: Effect of pH on the Formation, Disintegration and Antioxidant Activity of Mung Bean Protein Fibrils
Source: Antioxidants (Basel). 2025 Nov 25;14(12):1399. doi: 10.3390/antiox14121399 (PMC12729590; doi:10.3390/antiox14121399)

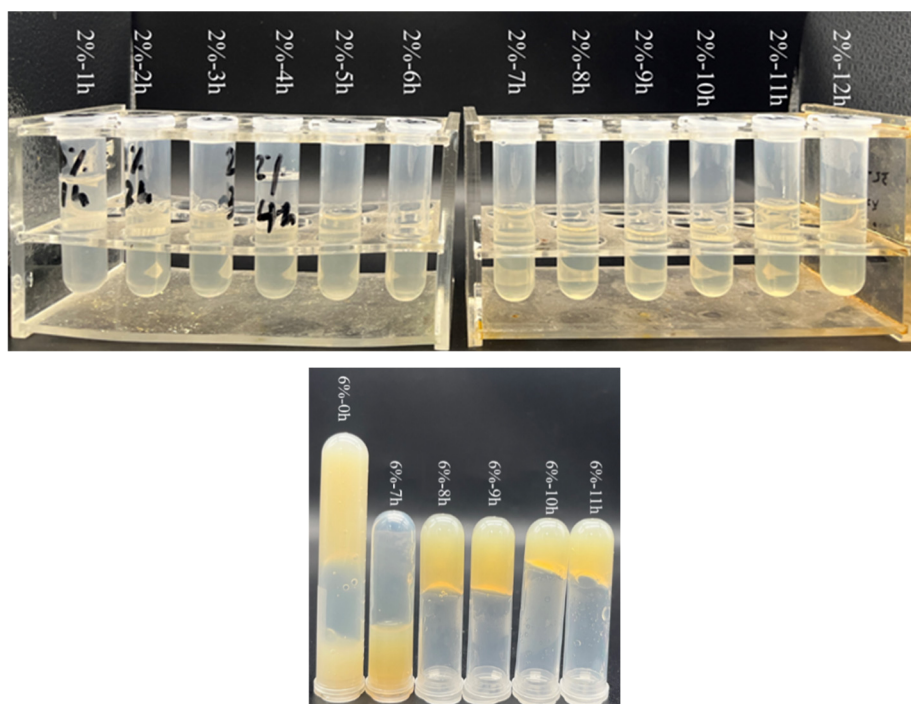

Figure S1 Visual observation of 2% (top) and 6% (down) MBP at pH 2.0 during heating treatment.

Supplement: Supplementary file 1 [file antioxidants-14-01399-s001.zip › antioxidants-3986864-supplementary.pdf]
